# Supplementary material for: Effects of (–)-Loliolide against Fine Dust Preconditioned Keratinocyte Media-Induced Dermal Fibroblast Inflammation
Source: Antioxidants (Basel). 2021 Apr 26;10(5):675. doi: 10.3390/antiox10050675 (PMC8144948; doi:10.3390/antiox10050675)
Supplement: Supplementary file 1 [file antioxidants-10-00675-s001.zip › antioxidants-1209541-supplementary.pdf]

Supplementary data

Article: Effects of (–)-loliolide against fine dust preconditioned keratinocyte media-induced dermal fibroblast inflammation

Journal: Antioxidants

Authors: Ilekuttige Priyan Shanura Fernando, Mawalle Kankanamge Hasitha Madhawa Dias, Dissanayaka Mudiyanselage Dinesh Madusanka, Hyun-Soo Kim, Eui Jeong Han, Min Ju Kim, and Ginnae Ahn

HTT priming of FD-stimulated keratinocytes roleplay in attenuating intracellular ROS levels in preconditioned media treated HDFs

In compliance with Appendix A, HPM-FD treatment promptly increased intracellular ROS levels (indicated by higher fluorescence intensity) compared to the control and HPM treated groups. A minor increase of green fluorescence was observed in HPM treated HDFs compared to non-treated control group. ROS levels in HDFs were reduced when preconditioned media was obtained from HTT primed FD-stimulated HaCaT cells, anti-parallel to HTT dose.

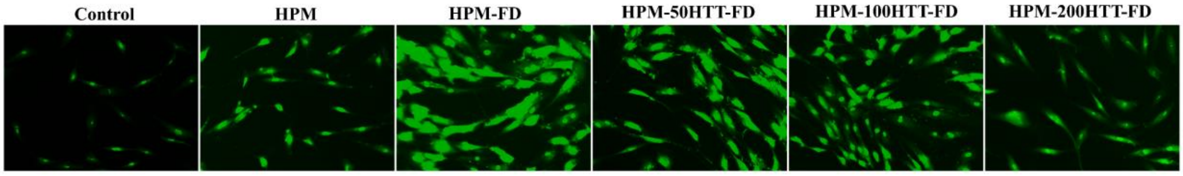

Figure S1

Evaluation of intracellular ROS levels in HDFs by fluorescence microscopy. HDFs were stimulated for 2 h with preconditioned media from FD-stimulated HaCaT keratinocytes with and without HTT pre-treatment. Intracellular ROS levels were measured 2 h after the stimulation period. Cell viability was evaluated after 24 h. Evaluations were carried out in triplicates (n=3) to ensure repeatability.

HTT priming of FD-stimulated keratinocytes roleplay in attenuating mitochondria depolarization in preconditioned media treated HDFs

Analysis was carried out using JC-1, a probe dye that undergo potential dependent accumulation in mitochondria emitting green (~529 nm) and red (~590 nm) fluorescence consecutively for its monomeric and aggregated forms. The decrease in red/green fluorescence intensity ratio indicates depolarized mitochondria. Most control cells showed a higher red fluorescence, while the majority of HDFs treated with CCCP, a commercial mitochondrial membrane disruptor, showed a higher green fluorescence. HPM-FD treatment of the HDFs showed minor mitochondrial damage, which was suppressed when treated with preconditioned media obtained from HTT primed, FD-stimulated HaCaT cells (HPM-HTT-FD).

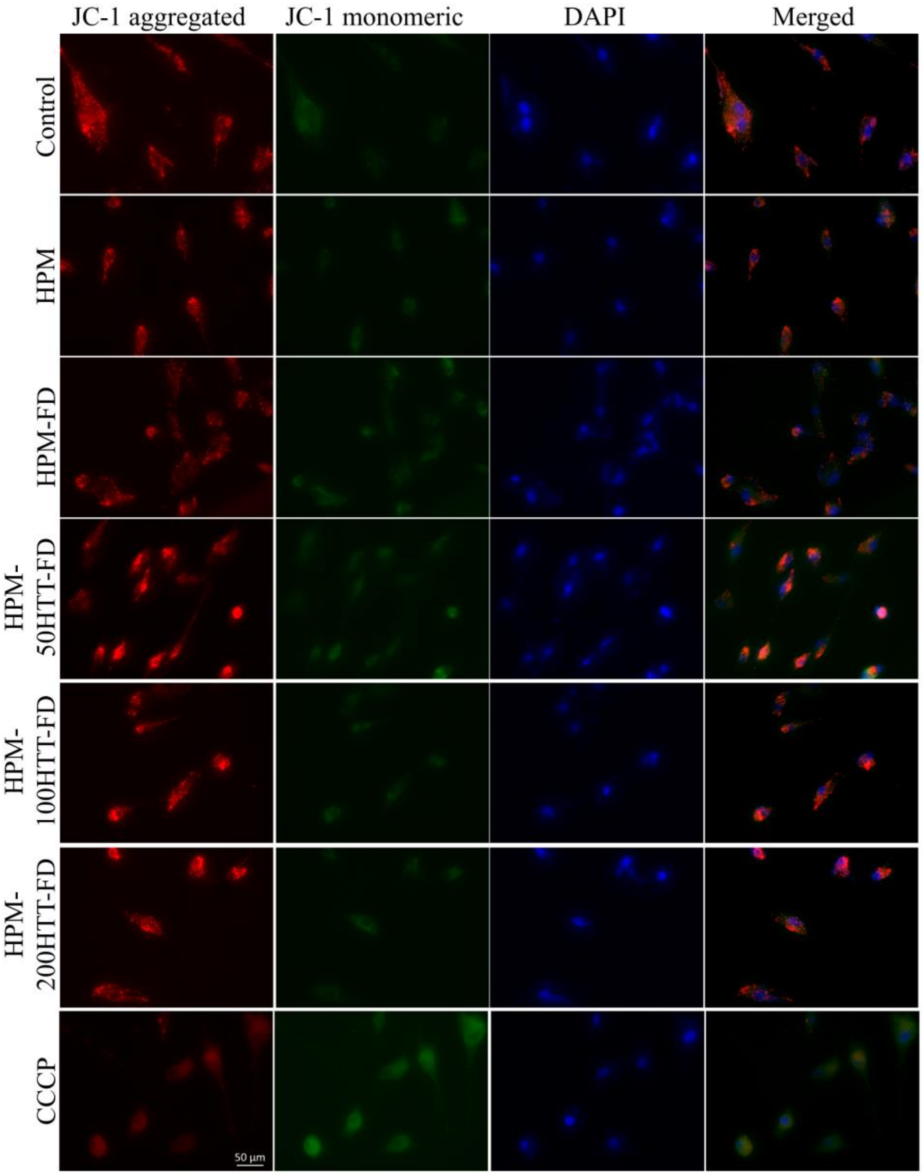

Figure S2

Fluorescence microscopic JC-1 assay analysis of mitochondria depolarization in HDFs treated with FD-stimulated HaCaT keratinocytes with and without HTT pre-treatment.
